# Effect of Carboxymethyl Cellulose and Polyvinyl Alcohol on the Dispersibility and Chemical Functional Group of Nonwoven Fabrics Composed of Recycled Carbon Fibers

**DOI:** 10.3390/ma17174209

**Published:** 2024-08-26

**Authors:** Kyungeun Kim, Gyungha Kim, Daeup Kim

**Affiliations:** Carbon & Light Materials Group, Korea Institute of Industrial Technology, Jeonju-si 54853, Republic of Korea; kke@kitech.re.kr (K.K.); gyungha@kitech.re.kr (G.K.)

**Keywords:** recycled carbon fiber, nonwoven fabrics, dispersibility, oxygen-containing functional group, mechanism

## Abstract

In this study, recycled carbon fibers (rCFs) recovered from waste carbon composites were used to manufacture wet-laid nonwoven fabrics. The aim was to improve dispersibility by investigating the changes in the dispersibility of carbon fibers (CFs) based on the content of the dispersant carboxymethyl cellulose (CMC) and the binder polyvinyl alcohol (PVA), and the length and basis weight of the CFs. In addition, the chemical property changes and oxygen functional group mechanisms based on the content of the CMC dispersant and PVA binder were investigated. The nonwoven fabrics made with desized CFs exhibited significantly improved dispersibility. For nonwoven fabrics produced with a fixed binder PVA content of 10%, optimal dispersibility was achieved at a dispersant CMC concentration of 0.4%. When the dispersant CMC concentration was fixed at 0.4% and the binder PVA content at 10%, the best dispersibility was observed at a CF length of 3 mm, while the maximum tensile strength was achieved at a fiber length of 6 mm. Dispersibility remained almost consistent across different basis weights. As the dispersant CMC concentration increased from 0.2% to 0.6%, the oxygen functional groups, such as carbonyl group (C=O), lactone group (O=C-O), and natrium hydroxide (NaOH), also increased. However, hydroxyl group (C-O) decreased. Moreover, the contact angle decreased, while the surface free energy increased. On the other hand, when the dispersant CMC concentration was fixed at 0.4%, the optimal binder PVA content was found to be 3%. As the binder PVA content increased from 0% to 10%, the formation of hydrogen bonds between the CMC dispersant and the PVA binder led to an increase in C=O and O=C-O bonds, while C-O and NaOH decreased. As the amount of oxygen increased, the contact angle decreased and the surface free energy increased.

## 1. Introduction

Carbon fiber (CF) is a material characterized by high specific strength, low density, and chemical stability, due to which it is actively utilized in high-tech industries, such as aviation, space, and wind power [1,2,3,4,5,6,7,8,9], while carbon composites are in the spotlight as lightweight materials that are likely to be integral to all industries in the future [10,11,12,13,14,15,16]. However, due to the high price and expensive manufacturing process of CFs, there are certain limitations to the application of carbon composites to components in mass industries, such as automobile parts. Furthermore, carbon composites are disposed of in landfills and incinerated after their functional life expires, thereby damaging the environment by causing pollution. To address this issue, developing upcycling technology to recover and recycle carbon fibers (rCFs) from waste carbon composites is crucial [17,18,19].

Methods for generating carbon composites include hand lay-up, filament winding, autoclave, pultrusion, and compression molding. When using rCFs, employing compression molding to create nonwoven fabrics offers several advantages, such as facility cost and processing convenience. As a result, it is considered the most efficient method for commercialization purposes [19,20,21]. Nonwoven fabrics, which are an intermediate material used in the compression molding of CFs, can be classified into dry and wet methods. However, according to previous research that implemented the dry method to create nonwoven fabrics in air, distortions were reported when weaving rCFs, which were not flat like commercial nonwoven fabrics but formed layers that resulted in defects [22]. Furthermore, in the spinning process conducted to improve the alignment of CFs, the yarn axis often deviates from the loading direction. Limitations in productivity have also been reported [23]. Moreover, considering that rCFs are short and discontinuous, with lengths of 0.1~60 mm, manufacturing nonwoven fabrics using the dry method, which continuously uses fibers of a certain length or more, has been considered a difficult task [24]. In contrast, when manufacturing nonwoven fabrics using the wet method, the fibers disperse in the water.

Notably, dispersibility has a decisive influence on the physical properties and quality of the final product. Therefore, when manufacturing nonwoven fabrics, a dispersant that improves the dispersibility of CFs and a binder that imparts adhesion and bonding strength between fibers to ultimately form a web are added. Hu et al., in their research paper on wet-laid nonwoven fabrics, reported that the introduction of a dispersant evenly disperses rCFs in nonwoven fabrics, thus increasing the interconnectivity between fibers while also improving electrical conductivity and the electromagnetic wave shielding function [25]. However, the addition of excessive dispersants may create multiple holes during the nonwoven fabric manufacturing process [26]. Furthermore, it has been reported that upon reaching a certain concentration of dispersant, minimal change occurs in the dispersibility of nonwoven fabrics even when the content is increased [27]. Another study found that the interlaminar fracture strength of composites made of nonwoven fabrics using a cross-linked styrene/acrylic (xSA) binder increased up to 89% compared with composites without any binder added to it, indicating that a binder not only increases the bonding strength between fibers but also affects the adhesion of fibers and resins [28]. However, the excessive addition of the binder acts as a barrier that blocks the pores between fibers, resulting in incomplete impregnation of nonwoven fabrics and resin when manufacturing composite materials [29]. This, in turn, results in the degradation of the physical properties and quality of the final product. Therefore, to optimize the physical properties of carbon composites using nonwoven fabrics manufactured through a wet process, it is necessary to establish optimal conditions for the dispersants and binders added to improve the dispersibility of CFs in nonwoven fabrics. Notably, existing research has significantly explored the optimal conditions for the dispersants and binders added to improve the dispersibility of CFs when manufacturing CFs wet nonwoven fabrics and has also evaluated the properties of carbon composites. However, investigations into the mechanism of change in the surface properties and chemical structures based on dispersant and binder-added CFs is scarce.

In this study, optimal conditions for manufacturing wet nonwoven fabrics using rCFs were derived based on the contents of the dispersant and the binder utilized to improve the dispersibility of CFs. Furthermore, dispersibility with regard to the length and basis weight of the CFs was examined. In addition, changes in the chemical state of CFs and the oxygen functional group mechanism in relation to the contents of the dispersant and the binder were investigated.

## 2. Materials and Experimental Procedure

### 2.1. Materials

**Figure 1:**
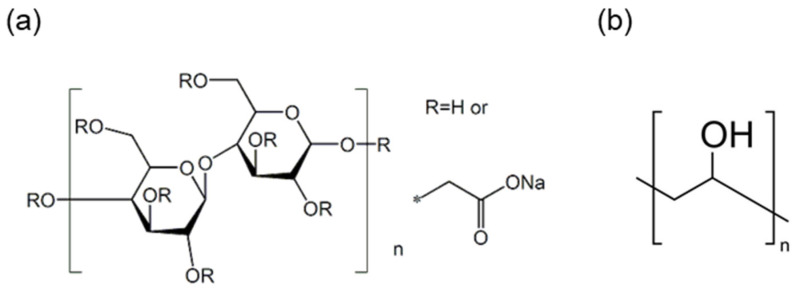
Chemical structure of (**a**) carboxymethyl cellulose and (**b**) polyvinyl alcohol.

This study used rCFs recovered from a T700-grade automobile fuel tank manufactured by Toray (Japan). Table 1 presents the physical properties of these CFs. Notably, acetone (99.5%) was used to desize the fibers, while the dispersant and binder added to the wet-laid nonwoven fabrics were carboxymethyl cellulose (CMC) (Figure 1a) and polyvinyl alcohol (PVA) (Figure 1b), respectively. Figure 1 depicts the chemical structures of the dispersant and the binder.

### 2.2. Experimental Methods and Analysis of Characteristics

**Figure 2:**
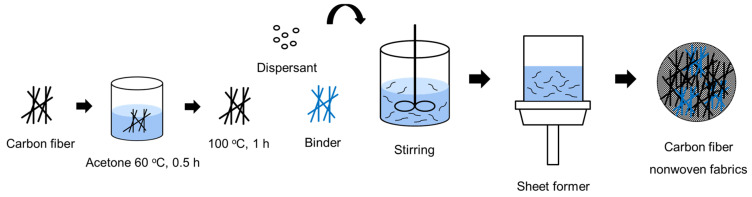
Schematic diagram of the wet nonwoven fabric manufacturing method in this study.

To recover CFs from the carbon composites used in automobile fuel tanks, the impurities and sizing agents remaining on the surface of the CFs had to be completely removed. Therefore, the fibers were immersed in acetone solution for 2 h, washed three times with acetone, and then dried at 100 °C for 1 h. Subsequently, 2 to 8 g of dispersant was dissolved in 500 to 2000 mL of distilled water using a stirrer for 180 min at a stirring speed of 1000 rpm. Next, 0.5 to 2 g of CFs, 0.05 to 0.2 g of binder, and 600 mL of distilled water were added to the dispersion solution and stirred for 1 to 20 min. This mixed solution was then used to produce nonwoven fabrics using a sheet former. The manufactured nonwoven fabric was dried at 80 °C for 120 min. Furthermore, the dispersant solution was prepared by varying the concentration of the dispersant in the range of 0.2~0.6%, while the amount of binder added was in the range of 0.5~10%. A schematic diagram of the manufacturing process for wet-laid nonwoven fabric is illustrated in Figure 2.

As for the CFs, the quantity of impurities and sizing agent on the fiber surface obtained through desizing was analyzed using thermogravimetric analysis (TGA, WATERS, model: discovery SDT 650, USA), while the mass change of the sample was examined by increasing the temperature to 1000 °C at a temperature increase rate of 10 °C/min in a nitrogen atmosphere. Each condition was conducted at least 3 times to attain the average value. To evaluate the dispersibility in terms of the conditions for nonwoven fabric manufacturing, a formation measuring analyzer (OpTest, model: LPA07, Canada) was employed to make evaluations relative to the value obtained when the dispersion of mass-produced nonwoven fabrics was set to 100%, with the average value obtained by carrying out the measurements 5 times for each condition. A formation measuring analyzer is equipment that analyzes the formation (bonding) of nonwoven fabrics, paper, film, etc., through image analysis using a charge-coupled (CCD) camera. It measures the uniformity of fiber bonding by analyzing images obtained using light intensity. The device provides a relative value that indicates how uniformly the fibers are dispersed compared to a reference sample (a value higher than 1 indicates relatively uniform dispersion, while a lower value indicates non-uniform dispersion). Furthermore, the mechanical properties of nonwoven fabrics were investigated by conducting a tensile test (Instron, model: 5567, USA) in accordance with the KS K ISO 9073-18 standard [30], with the average value attained on performing the test more than 5 times per test condition. Furthermore, to analyze changes in the chemical functional groups on the surface of the carbon fibers with regard to the content of the dispersant and the binder, X-ray photoelectron spectroscopy (XPS, Thermo Fisher Scientific, model: Nexsa, USA) was employed to carry out chemical analysis under the conditions of a passing energy of 50 eV and a beam size of 400 μm. In addition, to analyze the changes in surface energy, dynamic contact angle measurements were necessary. The contact angles were measured using the Wilhelmy plate method according to ASTM D1331–20 standards. Hydrophilic water and hydrophobic diiodomethane (99.9%, Sigma–Aldrich Co., LLC., USA) were used, with a dispensing speed of 6 mm/min. The contact angles for each liquid were measured, and the average values were calculated. Each test condition was measured more than 20 times, and the average contact angles were used to calculate the surface energy.

## 3. Results and Discussion

### 3.1. Changes in Dispersibility and Chemical Properties Based on the Concentration of the Dispersant

**Figure 3:**
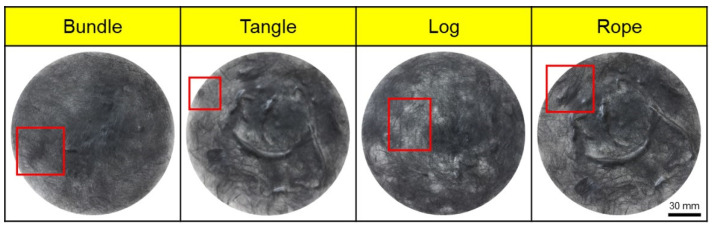
Types of defects in nonwoven fabrics. (red squares: main defect of nonwoven fabrics).

**Figure 4:**
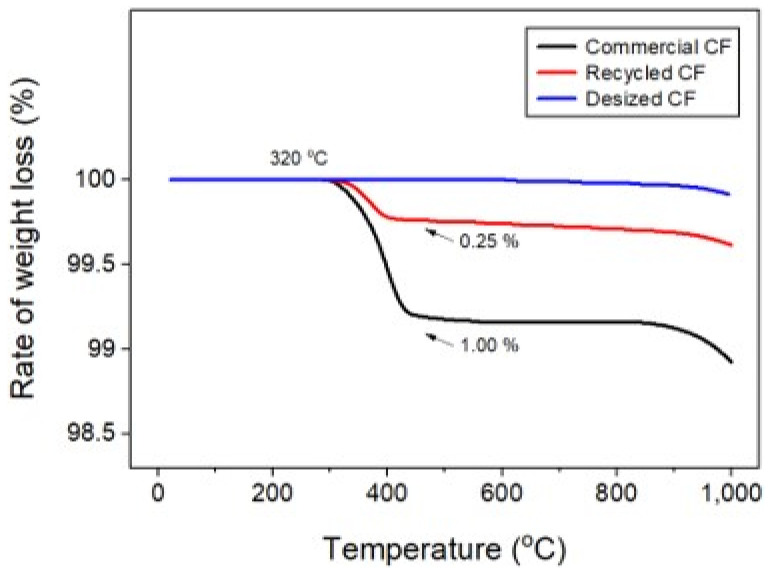
Thermogravimetric analysis of carbon fibers.

Poor dispersibility of wet nonwoven fabrics is responsible for various defects, such as bundle, tangle, log, and rope (Figure 3), where CFs are lumped into bundles. Therefore, experiments were conducted to address these defects and improve dispersibility. In this study, the dispersibility of CF-based nonwoven fabrics was evaluated using a formation measuring machine, with the dispersion index set to 1. Notably, mass-produced nonwoven fabrics are considered the standard for dispersibility evaluation. A value of 1 or more meant that the dispersibility was better than that of mass-produced nonwoven fabrics, while a value of 1 or less meaning reduced dispersibility (Ref. indicates the dispersion when mass-produced nonwoven fabric was used). The quantity of impurities and sizing agents on the surface of the CFs was confirmed using TGA, as shown in Figure 4. In this study, even a small weight change was interpreted as significant, as it aimed to assess the weight changes of epoxy resin and impurities remaining on the surface of the CFs. In the case of commercial CFs, a weight loss of approximately 1% was observed, which can be attributed to the weight change caused by the removal of the sizing agent coated on the fiber surface. In contrast, the recovered and rCFs exhibited a weight loss of approximately 0.25%, which may be a result of the chemical decomposition treatment conducted for the waste CF composite material, as the chemicals used in the process of separating the CFs may have remained on their surface. Notably, no change in weight was observed for the CFs desized using acetone, confirming that impurities and sizing agents on the surface of these CFs were completely removed by the desizing treatment. 

**Figure 5:**
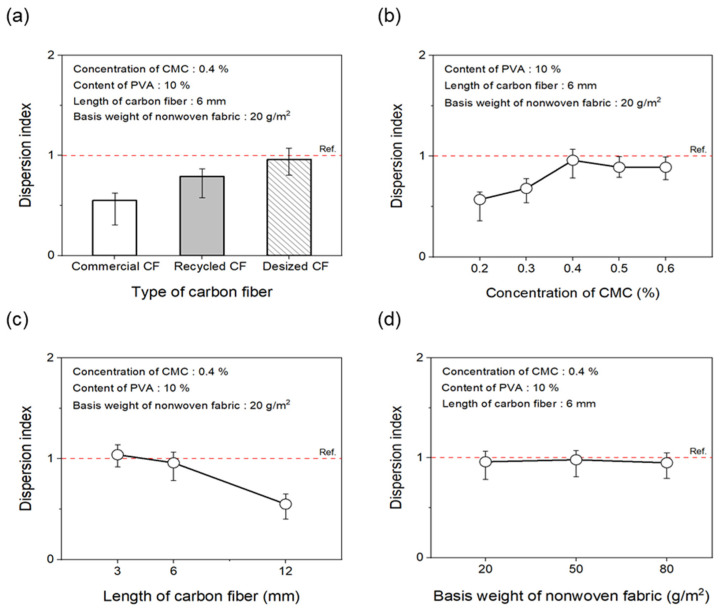
Dispersion index of nonwoven fabrics according to manufacturing conditions: (**a**) type of carbon fibers, (**b**) concentration of CMC, (**c**) length of carbon fibers, (**d**) basis weight of nonwoven fabrics.

**Figure 6:**
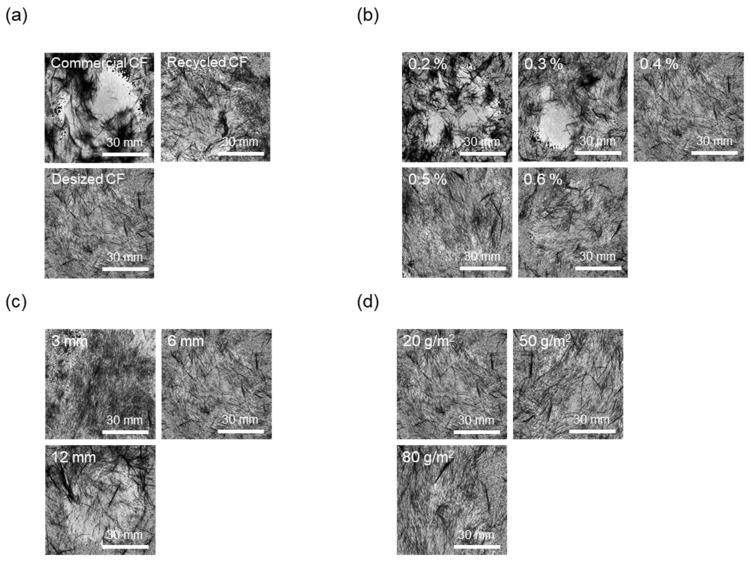
Dispersibility evaluation images of nonwoven fabrics according to manufacturing conditions: (**a**) type of carbon fibers, (**b**) concentration of CMC, (**c**) length of carbon fibers, (**d**) basis weight of nonwoven fabrics.

As shown in Figure 5a and Figure 6a, to evaluate the effect of the desizing treatment on the dispersibility of CF nonwoven fabrics manufactured using different types of CFs, the concentration of the dispersant CMC was fixed at 0.4%, and that of the binder PVA was fixed at 10%. In the case of nonwoven fabrics made from commercial CFs, the dispersion index was found to be 0.55. This result may be due to the sizing agent coating the surface of the CFs, which makes the fibers clump together instead of individually dispersing in water, yielding low dispersibility. In addition, since the epoxy sizing agent was removed from the rCFs due to the chemical decomposition treatment during the recovery process, the dispersion index of the CFs was 0.79, possibly due to the influence of chemicals remaining on the fiber surface during the decomposition process. Interestingly, when the rCFs was desized, the dispersion index of the nonwoven fabrics was 0.96, indicating a dispersibility that was 70% and around 20% higher than commercial CFs and rCFs, respectively. This was achieved as a result of the desizing treatment, which completely removed the impurities and sizing agents from the surface of the CFs, thereby allowing their individual dispersion and gradually improving the agglomeration phenomenon. This further contributed to the improved dispersibility of the CFs in nonwoven fabrics. 

According to Kim et al., desizing treatment is a fundamental process involved in manufacturing wet nonwoven fabrics, as desized CFs show improved wettability with fluids compared with those coated with a sizing agent, thereby improving the dispersion of fibers in water [31]. In this study, when manufacturing CF nonwovens through the wet process, it was determined that using CFs that had the chemicals on their surface completely removed through desizing treatment improved the dispersibility of the nonwovens. As a result, all subsequent experiments were conducted using materials that had undergone the desizing process. 

To evaluate the dispersibility in terms of the concentration of the dispersant added to improve the dispersibility of CFs, the content of the binder PVA was fixed at 10%. Subsequently, nonwoven fabrics were manufactured using different concentrations of the dispersant CMC, the results of which are shown in Figure 5b and Figure 6b. When manufacturing nonwoven fabrics with the concentration of dispersant CMC being 0.2% to 0.6%, the dispersion index increased until the concentration reached 0.4%, but then tended to decrease slightly at concentrations of 0.5% and 0.6%. Notably, at concentrations of 0.2% and 0.3%, the CFs were not uniformly dispersed, showing low dispersion indices of 0.57 and 0.68, respectively, along with empty areas observed in several places. This finding may be attributed to the insufficient amount of dispersant, which hindered the dispersibility of the CFs. As a result, the fibers had difficulty unraveling, in turn causing a decline in formability when manufacturing composite materials. Notably, the dispersibility of the CFs improved at a concentration of 0.4%, showing a dispersion index of 0.96. Furthermore, partially non-uniform parts of CFs were observed at concentrations of 0.5% and 0.6%, along with a dispersion index of 0.89, indicating slightly lowered dispersibility. Through these experiments, 0.4% was determined as the optimal concentration for manufacturing nonwoven fabrics using CMC as the dispersant. Therefore, for subsequent experiments, the concentration of the dispersant CMC was fixed at 0.4%. The dispersibility of the nonwoven fabrics was also evaluated by considering the length of the CFs as a variable (Figure 5c and Figure 6c) to find that it tended to decrease as the length of the CFs increased up to 12 mm. In particular, nonwoven fabrics composed of CFs with lengths of 3 mm and 6 mm showed excellent dispersibility as the CFs dispersed uniformly without leaving any empty sections, attaining dispersion indices of 1.13 and 0.96, respectively. In contrast, with regard to the nonwoven fabrics manufactured using CFs of 12 mm length, defects were confirmed, with the fibers twisting and clumping together, along with a low dispersion index of 0.72. Overall, this investigation confirmed that, as the length of CFs increased, they became entangled and started forming defects, such as logs and ropes, thus reducing dispersibility. Therefore, the optimal length of CFs for improving dispersibility was considered to be 3 mm. Figure 5d and Figure 6d present the results of the dispersibility of the nonwoven fabrics based on the basis weight. A good dispersion index of 0.92 or more was achieved at the weight of 20~80 g/m^2^. At the basis weight of 80 g/m^2^, although it was difficult to accurately confirm the degree of dispersion due to the covering of CFs in nonwoven fabrics, the dispersibility was considered to be almost similar to that for the entire weight range noted above. 

**Figure 7:**
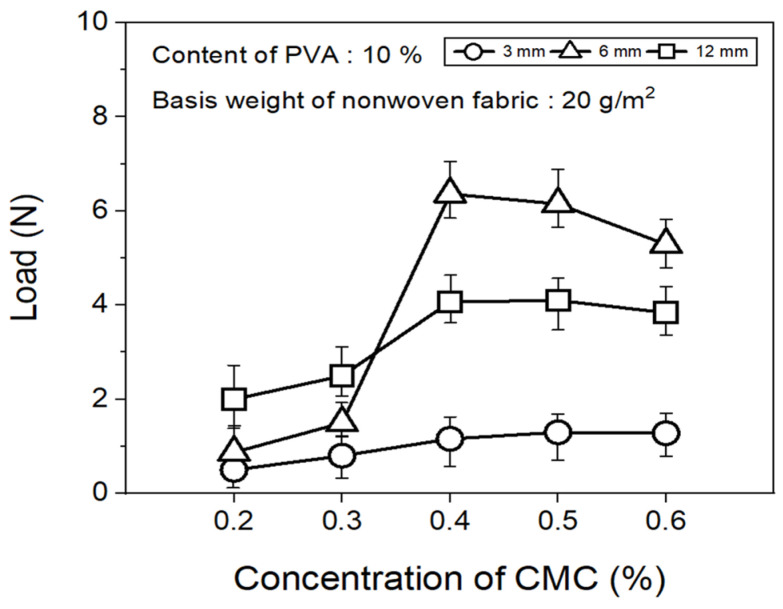
Tensile strength of nonwoven fabrics according to concentration of CMC.

**Figure 8:**
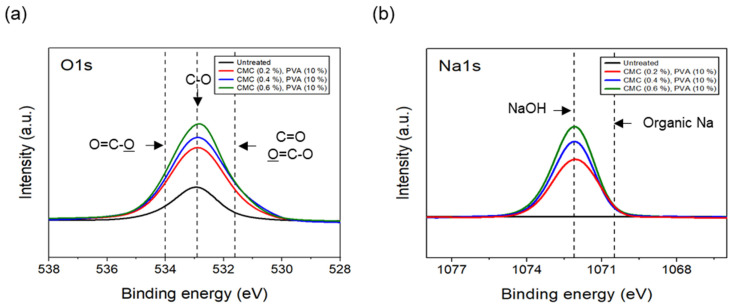
XPS spectra of carbon fibers covered with concentration of CMC: (**a**) O1s XPS spectra and (**b**) Na1s XPS spectra.

The tensile strength of the nonwoven fabrics, evaluated by fixing the binder PVA content at 10% and manufacturing nonwoven fabrics using different concentrations of dispersant CMC and lengths of CFs, exhibited a similar trend to that observed for dispersibility (Figure 7). The tensile strength of the nonwoven fabrics, considering a dispersant CMC concentration of 0.2~0.6%, increased up to 0.4% of dispersant concentration and then decreased slightly from a CMC concentration of 0.5%. In addition, when checking the tensile strength value with regard to the length of CFs at a dispersant concentration of 0.4%, the CFs with excellent dispersibility exhibited the lowest tensile strength value of 1.16 N at 3 mm length, which increased significantly to reach 6.36 N at 6 mm, and then decreased again to 4.07 N at 12 mm. The reason for these variations was that, since the bonding force between fibers was low during nonwoven fabric manufacturing using a short length of 3 mm for CFs, the fibers can be easily broken even with minimal force. Meanwhile, at a length of 12 mm, defects in the agglomeration and cohesion of fibers led to a decrease in the dispersibility of the nonwoven fabrics, and a decrease in tensile strength. Since the dispersibility of CFs in nonwoven fabrics was observed to be best for carbon fibers of 3 mm length but the tensile strength was highest at 6 mm, the optimal length for CFs to maintain the dispersibility and strength of nonwoven fabrics was determined as 6 mm. Figure 8 presents the results of the XPS analysis conducted to examine the changes in the chemical functional groups on the surface of the CFs based on the concentration of dispersant CMC, with the content of binder PVA fixed at 10%. According to the O1s spectra results, as the concentration of the dispersant CMC increased to 0.6%, the hydroxyl group (C-O) decreased, while the carbonyl group (C=O) and lactone group (O=C-O) continued to increase compared with the CFs without any added dispersant. In the Na1s spectra, a new formation of natrium hydroxide (NaOH) was observed in the CFs to which the dispersant CMC was added. The Na present at the end of the dispersant CMC reacted with the oxygen on the CF surface, forming NaOH. As the concentration of the dispersant CMC increased, the amount of NaOH increased, while the amount of organic Na decreased. In this context, Kim et al. reported that C-O, C=O, and O=C-O bonds decreased when the sizing agent on the surface of CFs was removed during desizing treatment [31], while Qui et al. reported that, on adding graphene oxide (GO) to CFs, CMC significantly increased the amount of both the carboxyl group (-COOH) and C-O [32]. These findings indicated that when the dispersant CMC was added to desized CFs, the C-O functional group on the surface of the fibers and the oxygen present in the dispersant CMC combined to increase the C=O and O=C-O bonds, while C-O decreased relatively. Furthermore, as the concentration of the dispersant CMC increased, the natrium present at the end of the dispersant CMC reacted with the oxygen on the surface of the CFs to increase the amount of NaOH. 

In addition, Table 2 summarizes the O/C, which quantitatively determined changes in the composition of the surface of the CFs with dispersant CMC added to them and the degree of increase in oxygen content based on the concentration of the dispersant CMC. Notably, when adding 0.6% dispersant CMC to the CFs, the amount of carbon and nitrogen declined compared with the CFs without any CMC, but the amounts of oxygen and natrium increased. Furthermore, the O/C, which also reflected the degree of interfacial bonding strength of the CFs, increased as the concentration of dispersant CMC increased to 0.6%. Meanwhile, CFs with 0.2% dispersant CMC showed a value of about 60% or more compared with CFs without dispersant CMC. In particular, at a concentration of 0.4%, O/C increased to 0.28, which was about twice that of the CFs without any dispersant CMC added. Moreover, this was higher than the surface activity standard of 0.14 [33]. Therefore, it was observed that, even when only 0.4% of the dispersant CMC was added to the fibers, the surface activity was excellent and the interfacial bonding strength was expected to improve.

**Figure 9:**
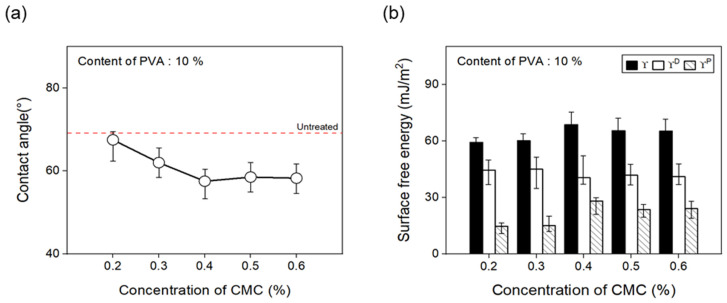
Contact angle and surface free energy of carbon fibers covered with concentration of CMC: (**a**) Contact angle and (**b**) surface free energy.

To confirm the change in the surface free energy of CFs in response to the concentration of dispersant CMC, the concentration was changed from 0.2% to 0.6%. Figure 9 presents the contact angles measured using hydrophilic and hydrophobic solutions. The polar and nonpolar surface free energy values were calculated using Equation (1), as noted below:

(1) $$\frac{\gamma_{L}(1+\cos\theta )}{2{\left(\gamma_{L}^{D}\right)}^{\frac{1}{2}}}={\left(\gamma_{S}^{P}\right)}^{\frac{1}{2}}\times {\left(\frac{\gamma_{L}^{P}}{\gamma_{L}^{D}}\right)}^{\frac{1}{2}}+{\left(\gamma_{S}^{D}\right)}^{\frac{1}{2}}$$

The contact angle of the CFs decreased when using a 0.4% concentration of the dispersant CMC compared with the CFs without any dispersant CMC, showing an almost similar trend from 0.4% to 0.6%. In addition, using Equation (1), the surface free energy ($\gamma_{L}$) was calculated from changes in the contact angle measurements of the surface of the CFs with regard to the concentration of dispersant CMC and the presence or absence of the binder PVA. Both polar free energy ($\gamma_{L}^{P}$) and nonpolar free energy ($\gamma_{L}^{D}$) were calculated. The classification results and polarity/surface energy ratio values are shown. The results showed that up to a 0.4% concentration of the dispersant CMC, the surface energy of the CFs increased, which was within the error range from 0.4% to 0.6%. Furthermore, the polarity/surface energy ratio achieved the highest value of about 33% at a 0.4% concentration of the dispersant CMC, while concentrations of 0.5% and 0.6% attained slightly decreased but similar results. Drawing on these findings, the optimal concentration of dispersant CMC was established to be 0.4%. Overall, it was observed that, as the CMC percentage increased, so did the amount of oxygen on the surface of the CFs and the polar surface free energy, while the contact angle decreased.

### 3.2. Changes in Dispersibility and Chemical Properties Based on the Content of the Binder

**Figure 10:**
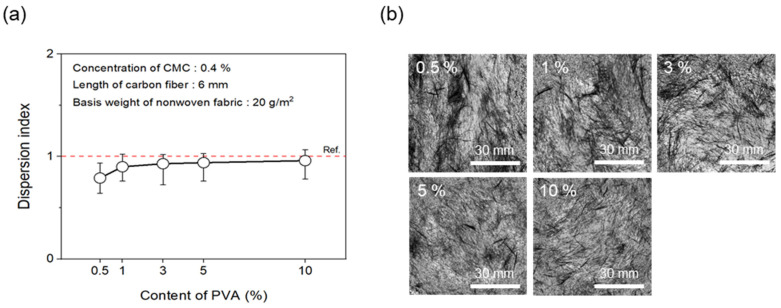
Dispersion index and dispersibility evaluation images of nonwoven fabrics according to content of PVA: (**a**) Dispersion index and (**b**) dispersibility evaluation images.

To evaluate dispersibility in terms of the content of binder added to improve the bonding strength between CFs, wet-laid nonwoven fabrics were manufactured by adding the binder PVA to the dispersant CMC. First, the concentration of the dispersant CMC was fixed at 0.4%. Figure 10 shows the results of the dispersion index and photos of the dispersibility evaluation for nonwoven fabrics with regard to various contents of the binder PVA. Notably, the dispersibility of the nonwoven fabrics showed an almost similar trend in response to the addition of binder PVA above a certain level. At 0.5% binder PVA content, a dispersion index of only 0.79 was observed. However, from 1% to 10%, substantial dispersibility was observed, with a dispersion index of over 0.90. Notably, even when only 0.5% of the binder PVA content was added, the dimensional stability of the nonwoven fabrics was found to be largely good. This phenomenon likely played a role in increasing bonding strength by filling the voids between CFs since the binder PVA melted during the drying process of the nonwoven fabrics. In addition, a level of hardness that was similar to mass-produced nonwoven fabrics was confirmed at 3% binder PVA content, which indicated sufficient dispersibility and hardness at less than 10% PVA content. Therefore, the optimal PVA content was considered to be 3%. 

**Figure 11:**
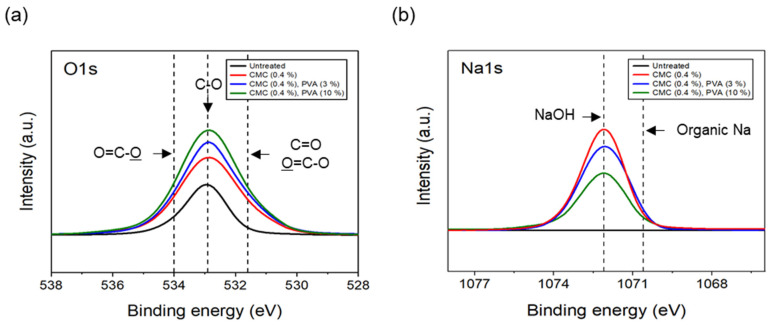
XPS spectra of carbon fibers covered with content of PVA: (**a**) O1s XPS spectra and (**b**) Na1s XPS spectra.

The results of the XPS analysis conducted on the CFs considering different quantities of binder PVA at a fixed concentration of 0.4% dispersant CMC are shown in Figure 11. The O1s spectra results showed that, as the content of binder PVA increased to 10%, C=O and O=C-O of the CFs gradually increased, while C-O decreased relatively, compared with the surface of the CFs without binder PVA. Furthermore, in the case of Na1s spectra, as the content of binder PVA increased to 10%, the bonding of NaOH formed on the surface of the CFs with only the dispersant CMC tending to continuously decrease, while the amount of organic Na decreased slightly. This indicated that, when binder PVA was added to dispersant CMC, the carbon and oxygen present in the former combined with the oxygen and carbon on the surface of the CFs, thus increasing the bonds of C=O and O=C-O. Additionally, it was believed that the oxygen present in the dispersants PVA and CMC bonded with the CF surface, resulting in a relative decrease in C-O. Notably, since the hydroxyl group (-OH) bonded to the oxygen atom on the surface of CFs through a hydrogen bond with the oxygen atom of the dispersant CMC [23], a bond was observed between the natrium atom present at the end of the dispersant CMC and the oxygen on the surface of the CFs. Notably, the amount of NaOH and natrium decreased when the bond broke. 

Table 3 summarizes the O/C, which quantitatively determined the change in the composition of the surface of the CFs with dispersant CMC added to them and the degree of increase in oxygen content based on the content of the binder PVA. Compared with the surface of the CFs without the dispersant CMC and binder PVA, the amounts of carbon and nitrogen on the surface of the CFs with the added dispersant CMC and binder PVA decreased, but the amounts of oxygen and natrium increased. In particular, after adding up to 10% binder PVA, the amounts of carbon, nitrogen, and natrium decreased, but that of oxygen increased compared with the surface of the CFs without binder PVA. Therefore, the ratio of O/C increased at 10% binder PVA content. In particular, 3% of the binder PVA content increased the ratio by about 80% compared with the CFs without any binder PVA. Therefore, it was found that interfacial bonding strength would improve even when using only 3% binder PVA content, considering that the ratio remained within the error range when the content of the binder PVA was increased to 10%. 

**Figure 12:**
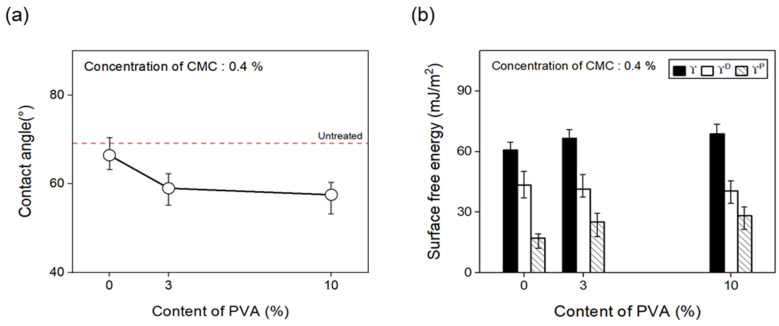
Contact angle and surface free energy of carbon fibers covered with content of PVA: (**a**) Contact angle and (**b**) surface free energy.

Furthermore, to confirm the change in surface free energy of the CFs with regard to the content of binder PVA, the contact angle was measured as shown in Figure 12, and the polar and nonpolar surface free energy values were calculated by substituting Equation (1) in Section 3.1. With the concentration of the dispersant CMC fixed at 0.4%, the surface energy of the CFs was observed to increase as binder PVA content of up to 10% was added, compared with the CFs without any addition of the binder PVA. The polarity/surface energy ratio of the CFs also increased by about 15%. This finding may be attributed to the gradual increase in the amount of C=O and O=C-O on the surface of the CFs due to the addition of the binder PVA, which decreased the contact angle and increased the polar free energy.

### 3.3. Changes in Functional Mechanisms Based on the Concentration of the Dispersant and the Content of the Binder

**Figure 13:**
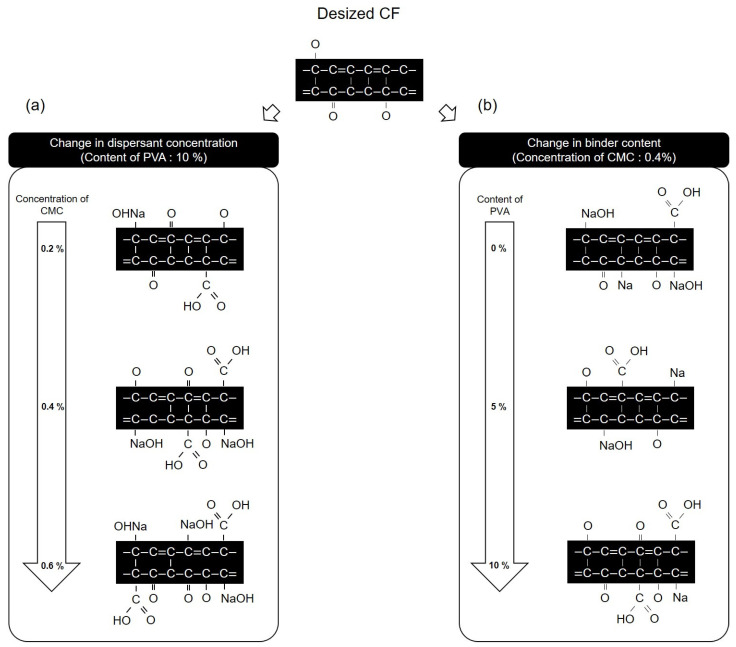
Schematic of the reaction of carbon fibers according to concentration of CMC and content: (**a**) Change in dispersant concentration and (**b**) change in binder content.

Figure 13 presents a schematic diagram of the chemical structure and oxygen functional group mechanism of the CFs surface, as observed from the results obtained on evaluating the dispersibility of CFs nonwoven fabrics manufactured using varying concentrations of dispersant and in the presence or absence of the binder. Figure 13a shows that when the concentration of the dispersant CMC was changed to 0.2 and the binder PVA content was fixed at 10%, the carbon and oxygen atoms in the dispersant CMC combined with the C-O and -OH on the surface of the CFs, thus increasing the C=O and O=C-O, which were present at the ends of the dispersant CMC, while decreasing the C-O. Furthermore, the amount of NaOH increased due to the reaction of natrium atoms with oxygen on the surface of the CFs. As the concentration of the dispersant CMC increased to 0.6%, the amounts of C=O, O=C-O, and NaOH increased, while C-O decreased along with it. Meanwhile, when fixing the concentration of dispersant CMC at 0.4% and increasing the content of binder PVA from 0% to 10%, changes in functional groups on the surface of the CFs were observed (Figure 13b). The CF surface composed of added dispersant CMC but without the binder PVA showed an increase in C=O, O=C-O, and NaOH, while the C-O bond decreased as the carbon, oxygen, and natrium atoms of the dispersant CMC bonded to the surface of the CFs. Furthermore, when 3% binder PVA content was added, the carbon and oxygen atoms present in the binder PVA bonded to the C-O and -OH on the surface of the CFs, as a result of which the amounts of C=O and O=C-O increased, while C-O decreased compared with those for the CFs without any binder added. It further increased the -OH of the binder PVA, and the oxygen atom of the dispersant CMC formed a hydrogen bond [34], which bonded to the oxygen on the surface of the CFs, causing the natrium atom present at the end of the dispersant CMC to bond with the oxygen of the CFs. Upon breaking this bond, the amount of NaOH decreased. Moreover, when increasing the binder PVA content to 10%, the CFs with 3% binder PVA content exhibited higher increased C=O and O=C-O, and decreased amounts of C-O and NaOH.

## 4. Conclusions

This study aimed to improve the dispersibility of rCFs when manufacturing wet nonwoven fabrics. Dispersibility was evaluated by considering different concentrations of the dispersant CMC and binder PVA, and varying lengths, basis weights, and types of CFs. Furthermore, changes in the chemical state of CFs and in the oxygen functional group mechanism were identified. The results showed significant improvements in the dispersibility of nonwoven fabrics when using desized CFs. With regard to the dispersant CMC, the optimal dispersibility was confirmed at a concentration of 0.4%. In addition, while the dispersion index was highest for 3 mm long CFs, the tensile strength reached its maximum value at a 6 mm length. The dispersibility of nonwoven fabrics in terms of the basis weight of the CFs showed a similar trend, while a binder PVA content of 3% was found to be optimal. With regard to changes in chemical functional groups based on the concentration of the dispersant CMC, it was observed that as the concentration of the dispersant CMC increased from 0.2% to 0.6%, the reaction of the CMC and the surface of the CFs led to an increase in the oxygen functional groups, such as C=O, O=C-O, and NaOH, while C-O decreased. At the same time, the contact angle decreased, while the surface energy increased. Furthermore, with an increase in the content of binder PVA up to 10%, C=O and O=C-O increased due to hydrogen bonding between the dispersant CMC and binder PVA, while the amount of C-O and NaOH decreased. Furthermore, as the amount of oxygen increased, the contact angle decreased and the surface free energy increased. 

Notably, in our future studies, we plan to contribute to the commercialization of automobile parts by producing and evaluating rCF-based nonwoven fabrics manufactured under optimal conditions, and by generating and examining carbon composites made of thermoplastic resin and thermosetting resin.

## Figures and Tables

**Table 1:** Properties of recycled carbon fibers in this study.

Type	Recycled CF
Tensile strength (GPa)	3.95
Modulus (GPa)	258
Elongation (%)	2.38
Density (g/cm^3^)	1.80

**Table 2:** Surface element composition of carbon fibers treated according to CMC concentration.

Dispersant (CMC)	Binder (PVA)	Elemental Composition (at. %)				O/C
		Carbon	Oxygen	Nitrogen	Natrium	
Untreated		85.66	12.46	1.88	-	0.15
0.2%	10%	78.05	18.59	1.46	1.90	0.21
0.4%		75.00	21.10	1.26	2.64	0.28
0.6%		75.72	23.00	1.17	3.11	0.31

**Table 3:** Surface element composition of carbon fibers treated according to content of PVA.

Dispersant (CMC)	Binder (PVA)	Elemental Composition (at. %)				O/C
		Carbon	Oxygen	Nitrogen	Natrium	
Untreated		85.66	12.46	1.88	-	0.15
0.4%	0%	76.16	19.16	1.53	3.15	0.25
	3%	75.17	20.59	1.38	2.86	0.27
	10%	75.00	21.10	1.26	2.64	0.28

## Data Availability

The data presented in this study are available on request from the corresponding author.
